# A techno-economic analysis of diesel exhaust injection into mine tailings for carbon sequestration

**DOI:** 10.1016/j.heliyon.2024.e27791

**Published:** 2024-03-15

**Authors:** Durjoy Baidya, Gregory Dipple, Seyed Ali Ghoreishi-Madiseh

**Affiliations:** a Norman B. Keevil Institute of Mining Engineering, The University of British Columbia, Vancouver, BC, V6T 1Z4, Canada; b Department of Earth, Ocean and Atmospheric Science, The University of British Columbia, Vancouver, BC, V6T 1Z4, Canada

**Keywords:** Remote mines, Mine tailings, Carbon footprint, Energy optimization, Financial analysis, Decarbonized mining

## Abstract

The environmental impact of off-grid mines in remote, cold climates is significantly intensified by their dependence on fossil fuels for power and heating. A promising solution lies in the potential to capture and permanently store carbon within mine tailings, thus allowing the mining industry to take a leading role in carbon removal initiatives and provide sustainable solutions. This study explores energy-optimal design scenarios for flue gas injection into mine waste to capture carbon. The approach involves installing perforated pipes within dry stack tailings. The established reduced-order model in this research serves as a novel tool for decision-making, aiding in the selection of an appropriate perforation scheme for the injection pipes embedded in the tailings. A cost analysis is also performed to assess the financial viability of the proposed concept under different operating parameters. Operational expenses, particularly energy costs, are found to be influenced by the permeability of the tailings. In instances of lower permeabilities, larger injection pipes are required. The findings indicate that achieving viable operating costs for sequestering one tonne of carbon dioxide hinges on amenable pipe sizing and engineering. Additionally, the study estimates that maintaining a reasonable level (around 1%) between the power being decarbonized and the power required for the carbon sequestration operation is crucial.

## Nomenclature

**Abbreviations**
D: Dimensional
EHRS: Exhaust heat recovery system
FEA: Finite element analysis
IPL: Injection pressure loss
OPEX: Operating expenditure
RANS: Reynolds Averaged Navier-Stokes
ROM: Reduced-order model
TPL: Transportation pressure loss

**Symbols**
$A_{in}$: The surface area of the pipe inlet ($m^{2}$)
$A_{p}$: The total perforated surface area of the pipe ($m^{2}$)
$A_{r}$: The perforated surface area ratio of the injection pipe
$C_{P}$: Specific heat capacity (J/(kgK))
$K_{t}$: Permeability of the tailings ($m^{2}$)
$M_{{CO}_{2}}$: Amount of $CO_{2}$ sequestrated in a particular operating period (tonnes)
$M_{tailings}$: Quantity of tailings used in the operation in a particular operating period (tonnes)
$R_{wh}$: The width-to-height ratio of the tailings block
$R_{Ε}$: Energy ratio
$W_{comp}$: Energy consumer by the compressor operating (W)
$W_{fan}$: Energy consumer by the fan operating (W)
$c_{F}$: Forchheimer coefficient
$d_{h}$: Hydraulic diameter (m)
$f_{ui}$: Flow uniformity index
$n_{c}$: The number of perforations around the circumference of the injection pipe
${ox}_{elec}$: Piping cost to capture 1tonne of $CO_{2}$ in every operating year (C$)
${ox}_{piping}$: Electricity cost to capture 1tonne of $CO_{2}$ in every operating year (C$)
${ox}_{t}$: Total operating expenditure to capture 1tonne of $CO_{2}$ in every operating year (C$)
$p^{\prime }$: Mid-perforation pressure (Pa)
$p_{atm}$: Atmospheric pressure (Pa)
$r_{in}$: The inner radius of the injection pipe (m)
$r_{p}$: Perforation radius (m)
$t_{piping}$: Re-piping period (years)
${uc}_{elec}$: Cost of electricity (C$/kWh)
${uc}_{piping}$: The unit cost of piping ($/m)
$\eta_{comp}$: The efficiency of the compressor (%)
$\eta_{fan}$: The efficiency of the fan (%)
h: Height of the tailings block (m)
ΔQ: Outflow (m^3^/s)
L: Length of the injection pipe (m)
P: Pressure (Pa)
Q: Volumetric flow rate (m^3^/s)
RI: Reactivity index
Re: Reynolds number
i: Index
s: The center-to-center distance of the perforations (m)
w: Width of the tailings block (m)
γ: Adiabatic index
δ: Pipe roughness (m)
λ: Darcy-Weisbach friction coefficient
μ: Dynamic viscosity (Pa∙s)
ρ: Density $\left(\text{kg}/m^{3}\right)$

## 1 Introduction

Climate change encompasses the enduring alterations in temperature, precipitation, wind dynamics, and various components of the climate system of the earth, predominantly instigated by several human activities [1]. The impact of the increasing anthropogenic greenhouse gas emissions was concerning enough to declare climate action as one of the goals for sustainable development set by the United Nations [2]. A multi-faceted measure that incorporates creating public awareness, stringent regulations, reducing greenhouse gas emissions, and so forth must be implemented to fight climate emergencies. Of these, prioritizing the decline of fossil fuel emissions can be underscored as the foremost objective to avert further detrimental impacts on the climate [3]. Recognizing the magnitude of carbon emissions, a notable focus is being witnessed on the advancement of numerous innovative and sustainable carbon removal technologies along with several mitigation strategies. These approaches embrace direct air capture, enhanced weathering, afforestation, and bioenergy combined with carbon capture and storage [4]. With access to vast quantities of mafic and ultra-mafic tailings that possess substantial $CO_{2}$ capture and storage potential [5], the mining industry stands poised to play a pivotal role in spearheading efforts to combat climate change [[6], [7], [8]].

As an energy-intensive industry, mining bears its own responsibility to transition towards decarbonized operations [9]. Nonetheless, achieving a full-scale shift to renewable energy sources for power generation presents considerable challenges for the mining sector, particularly when expanding operations in off-grid areas to meet the growing demand for metals required in renewable energy technologies [10,11]. Due to their cold climatic locations, mines operating in remote areas depend heavily on fossil fuels, particularly diesel, for essential functions such as power generation and heating [11]. The injection of diesel flue gas from the power-generating units into mine tailings to capture and store $CO_{2}$ holds the potential to reduce the carbon footprint of those operations significantly [12].

Several studies highlighted the occurrence of passive carbonation in mine tailings, even under standard conditions, where the source of $CO_{2}$ is the ambient air [13,14]. Though the high surface area of tailings attained through the processing facilitates the carbonation process, the reaction rate can often be constrained due to the limitation in the availability of $CO_{2}$ [15,16]. Injection of $CO_{2}$-rich fluids into the tailings showed accelerated reaction rates in lab-scale experimental investigations [17,18]. Moreover, temperature on the higher end may positively influence the carbonation process in mine tailings [[19], [20], [21]]. These factors made the diesel exhaust injection into mine tailings an ideal candidate to decarbonize the mining operation in remote locations.

The attainment of large-scale carbon capture in mine tailings through flue gas injection successfully will not only mitigate the environmental footprint but also position mining as a significant player in the carbon market. However, it is essential to understand the underlying challenges for large-scale implementation of carbon capture in mine tailings through diesel exhaust injection and take the necessary steps to overcome those [22]. One of these challenges is the lack of financial analysis that can evaluate the economic viability of this concept. To bridge this knowledge gap, the present study further developed an existing framework (outlined in Ref. [12]) to devise an energy-optimal approach for flue gas injection into mine tailings for carbon sequestration. Drawing upon this framework, a comprehensive cost analysis was undertaken to assess the potential operational expenses linked with injecting diesel exhaust into mine waste for carbon capture. Moreover, a range of sensitivity analyses were performed on relevant operational parameters to gauge the potential variations in the performance of the concept proposed here from an economic standpoint.

## 2 Methodology

### 2.1 General model description

**Fig. 1:**
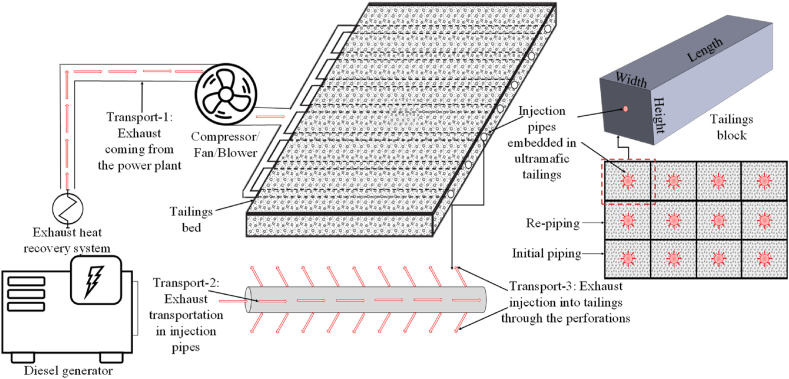
An illustration representing the proposed carbon sequestration approach tailored for off-grid mining sites (modified from Ref. [12]).

Installation of the perforated pipes in the series of equal-sized tailings blocks is the way to inject the diesel exhaust from the power plant operating in the remote, off-grid mines for carbon sequestration purposes. The perforated pipes, embedded in dry stack tailings, are assembled to maintain an optimum pressure and energy scenario throughout the injection process. The conceptual framework of the proposed approach is depicted in Fig. 1. This conceptual design requires the cooling of the exhaust by using an exhaust heat recovery system (EHRS) that was discussed thoroughly in Refs. [[23], [24], [25]]. Transport-1 denotes the transportation of the diesel exhaust from the EHRS to the tailings bed through a designated pipeline. The subsequent injection of this exhaust into an array of perforated pipes strategically situated within the deposited filtered tailings was prescribed. This arrangement ensures the consistent distribution of $CO_{2}$ throughout the system. The proposed framework involves partitioning the tailings into discrete blocks of identical dimensions, each featuring a central injection pipe enveloped by a specific volume of surrounding tailings. The dimensions of these blocks will be an operating decision depending on the permeability, reactivity capability, and availability of the tailings. These injection pipes will facilitate two concurrent processes: the conveyance of exhaust through the pipes (Transport-2) and its dispersion into the tailings through the pipe perforations (Transport-3). Upon the full reaction of a given tailings layer, a fresh layer can be established, accompanied by the introduction of a new set of injection pipes. The incorporation of a compressor, fan, or blower becomes crucial to enable the smooth flow of exhaust, effectively mitigating pressure drops incurred during the aforementioned transportation activities. This integration of the air handling unit is essential for propelling the exhaust into the tailings, thereby ensuring a consistent and uninterrupted discharge along the entire length of the pipe. Solid understanding and optimizing the pressure phenomena involved in Transport-1, 2, and 3 are critical for running the operation efficiently.

So far, a (1 + 1)D reduced-order model (ROM) was developed to predict the pressure profile for a perforated pipe embedded in a cylindrical block [12]. However, in practical scenarios, the tailings blocks will typically have a rectangular shape (illustrated in Fig. 1), necessitating the extension of the validated (1 + 1)D model to cater to the real-world application. In this study, this research gap was addressed by further extending the previously developed reduced-order model to accurately predict the fluid flow and the pressure profile during carbon sequestration in mine tailings with perforated injection pipes within a rectangular block scenario. The necessary accuracy of this extended (1 + 1)D ROM was attained by comparing the relevant outcomes form it with a commercially available finite element solver, COMSOL Multiphysics 6.1. Afterward, the (1 + 1)D ROM was coupled with a cost model developed here to estimate the variance in the operating expenditure (OPEX) to sequestrate 1tonne of CO_2_ at different operating and design constraints.

### 2.2 Extension of (1 + 1)D reduced-order model

For all the cases investigated here, the perforation scheme was selected based on the framework established and rigorously tested in Ref. [12]. In this configuration, the center-to-center distance of the perforations, known as pitch (s), was the same in both axial (along the length of the pipe) and radial (along the circumference of the pipe) directions. First, the number of perforations around the circumference ($n_{c}$) of the pipe was determined. Based on that, the pitch can be computed. Depending on the s and $n_{c}$, the number of the perforations in the axial direction ($n_{c}$) was determined. The size of the perforations ($r_{p}$) controlled the perforated surface area ratio ($A_{r})$ of the pipe.

#### 2.2.1 3D Numerical model development

**Fig. 2:**
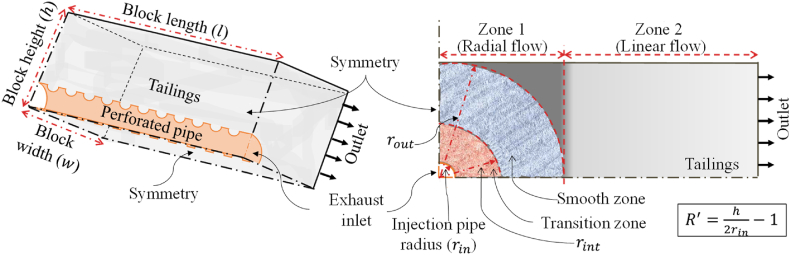
Diagram illustrating the computational domain of the rectangular tailings block for carbon sequestration, accompanied by the pertinent boundary conditions for the numerical modeling.

The 3D FE models were developed based on the numerical methodology that was previously established and validated in Ref. [12] for flue gas injection in mine tailings for carbon sequestration. The flow through the injection pipe (Transport-2), and the outflow through the perforation into the porous tailings (Transport-3) were modelled together based on their designated governing equations. The computational domains and the associated boundary conditions of the 3D FE models are shown in Fig. 2.

The Free and Porous Media Flow interface was activated in COMSOL Multiphysics 6.1. This allowed the flow in the free channel (through the pipe) governed by the Reynolds Averaged Navier-Stokes (RANS) equations. Equation (1) was the governing equation for the steady-state, turbulent, incompressible flow of exhaust, a Newtonian fluid, through the pipe, based on RANS formulations in terms of the time-averaged and varying components of the velocity field ($U_{i},u_{i}^{\prime }\;;\;i=1-3$).

(1) $$\rho_{exh}U_{j}\frac{\partial U_{i}}{\partial x_{j}}=\frac{\partial }{\partial x_{j}}\left[-p\delta_{ij}+\mu_{exh}\left(\frac{\partial U_{j}}{\partial x_{i}}+\frac{\partial U_{i}}{{\partial x}_{j}}\right)-\rho_{exh}\bar{u_{i}^{\prime }u_{j}^{\prime }}\right]$$

Here, $\rho_{exh}$, $\mu_{exh}$, and p denote the density and viscosity of the exhaust and associated pressure for the flow of exhaust through the pipe. The Reynolds stress tensor ($-\rho \bar{u_{i}^{\prime }u_{j}^{\prime }}$) in this equation indicates the measure of the turbulent fluctuations in the exhaust flow field. The next step was to solve the momentum equation, a critical exercise that required expressing the Reynolds stress tensor in relation to the time-averaged velocity vector components via the integration of a suitable turbulence model. The Algebraic yPlus model—a choice grounded in the well-established framework of Prandtl's mixing-length theory was employed as the turbulence model. This selection offered the advantage of both accuracy and computational efficiency. Notably, the Algebraic yPlus model exhibited robustness in the face of varying mesh sizes, mitigating sensitivity concerns that may arise in other modeling approaches. The investigations conducted here revealed that this model delivered noteworthy reductions in computational overhead, enhancing overall computational expediency and rendering it a favorable option for this study.

On the other hand, the flow in the tailings (porous domain) was governed by the Brinkman–Forchheimer equation, as mentioned in Refs. [26,27].

(2) $$\nabla p=-\frac{\mu_{exh}}{K_{t}}u+\bar{\mu_{exh}}\nabla^{2}u-c_{F}{K_{t}}^{-\frac{1}{2}}\rho_{exh}u\left|u\right|$$

This equation is composed of three distinct terms, each contributing to the comprehensive description of the fluid flow phenomena within the porous medium. The initial term, situated on the right-hand side, embodies Darcy's law, encapsulating the essential relationship between pressure gradient and flow rate. The proceeding term, an indicator of the Brinkman extension, encompasses the viscous influences operating within the porous medium, further refining the model's fidelity to real-world conditions. Intertwined with these components, the last term encapsulates the Forchheimer extension, an essential inclusion that effectively characterizes the high turbulent flow regime, as explained in prior research [28]. The effective viscosity at the wall, $\bar{\mu_{exh}}$ signifies a parameter conveniently assumed to be the same as $\mu_{exh}$, for the sake of modeling simplicity. The numerical simulations conducted here were found to have insignificant impact of the inertial forces. So, for the numerical environment considered here (where, ${Re}_{K}<1$), the Forchheimer constant ($c_{F}$) gravitated towards a value of zero ($c_{F}$ = 0).

A constant mass flow rate was integrated across the inlet boundary section (shown as exhaust inlet, $r_{in}$, in Fig. 2), aligned parallel to the boundary's normal direction, with the tangential velocity set to zero. Employing two symmetry planes allowed confining the analysis to one-quarter of the geometrical domain. This reduced the computational resource requirement while ensuring the integrity of the numerical analysis. Enforcing a no-slip condition at the top surface ensured that the fluid velocity relative to the wall velocity remained zero (u=0). A pressure outlet boundary condition was employed at the outlets, where the tangential stress components were prescribed to be zero. Continuity conditions were upheld at the perforated surfaces of the pipe, and a Dirichlet boundary condition was assigned at the interface between the fluid and the pipe. Due to its significantly smaller dimension compared to the overall geometry, the thickness of the pipe was deemed insignificant and consequently omitted in the simulation analysis. A rigorous grid independence study was conducted before commencing simulations using the newly formulated 3D finite element model. This meticulous investigation aimed to ascertain that the outcomes from the model remained impervious to alterations in the mesh configuration, ensuring the robustness and reliability of the subsequent analyses.

#### 2.2.2 (1 + 1) ROM development for tailings block

**Fig. 3:**
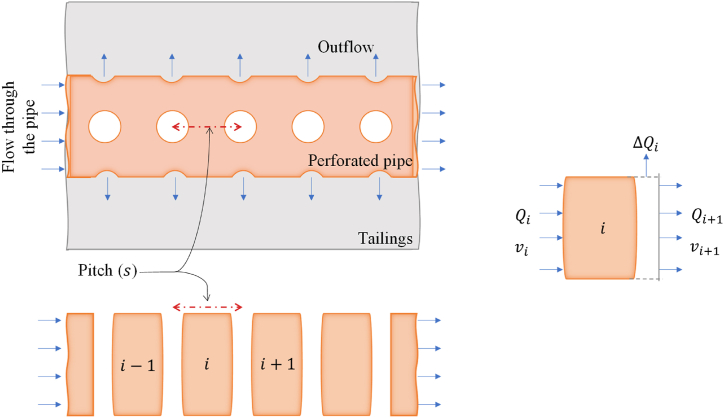
Development of the (1 + 1)D ROM for perforated injection pipes embedded in the rectangular tailings block (modified from Ref. [12]).

Following the methodology established in Ref. [12] for the (1 + 1)D ROM for cylindrical blocks, the whole length of the pipe, L, was partitioned into n number of segments having equal length of s. These segments were interconnected sequentially, where information flew from one segment to the next. Fig. 3 illustrates the discretization approach employed in the (1 + 1)D ROM for tailings block. $Q_{i}$ represents the quantity of exhaust conveyed as the inflow to the i ^th^ section, ${\Delta Q}_{i}$ signifies the exhaust outflow into the tailings from that specific perforated section, and $Q_{i+1}$ denotes the flow forwarded from the i ^th^ section to the immediately subsequent section.

The exhaust flow through the pipe resulted in a frictional pressure loss due to the friction with the inner surface of the pipe. This is denoted as ${{\Delta p}_{d}}_{i}$, and was estimated using the Darcy-Weisbach equation [29] mentioned in equation (3) for every discretized pipe segment. TPL (transportation pressure loss, ${{\Delta p}_{d}}_{i}$) here was a function of the Darcy-Weisbach friction coefficient ($\lambda_{i})$ [29]. It was iterated based on the Reynolds number (${Re}_{i})$ at every i ^th^ section by using equations (4), (5), respectively, for turbulent and laminar flow. Equation (6) was used to calculate ${Re}_{i}$ at every section of the discretization.

(3) $${{\Delta p}_{d}}_{i}=-\;s\left(\frac{\rho_{exh}}{{2d}_{h}}\right){\left(\frac{Q_{i}}{A_{in}}\right)}^{2}\lambda_{i}$$

(4) $$\lambda_{i}=0.0055\left\{1+{\left(2\times 10^{4}\frac{\delta }{2r_{in}}+\frac{10^{6}}{{Re}_{i}}\right)}^{\frac{1}{3}}\right\}$$

(5) $$\lambda_{i}=\frac{64}{{Re}_{i}}$$

(6) $${Re}_{i}=\frac{2r_{in}\rho_{exh}Q_{i}}{\mu_{exh}A_{in}}$$

where, the hydraulic diameter of the pipe, $d_{h}$, is equivalent to the actual diameter of the circular-shaped injection pipe. The absolute roughness and the inner radius of the injection pipe is signified as δ, and $r_{in}$, respectively. $A_{in}$ indicates the surface area of the pipe inlet.

The rectangular shape of the tailings block was considered partitioned into two zones, as shown in Fig. 2 with the dashed red lines. Darcy's law for radial flow was applied to the zone on the left (zone 1) assuming a cylinder originating from the center of the setup with a diameter that was equal to the height of the block. The zone on the right (zone 2) was presumed to have Darcy's law of linear flow active. It has already been proved in Ref. [12] that the arrangement of the perforations could impact the pressure profile by resulting in abrupt flow patterns in the porous domain very adjacent to the perforations. This region was denoted as transitions zone (shown in Fig. 2) and was found to expand to approximately half of the length of the pitch used. Subsequently, the flow evolves into a uniform pattern as it enters the smooth zone. The radial velocity of the exhaust in the transition and the smooth zone can be represented, respectively, with equations (7), (8).

(7) $$u_{r}^{\left(1\right)}\left(r\right)=\frac{Q}{2\pi rL}+\left[\frac{Q}{A_{p}}-\frac{Q}{2\pi rL}\right]\left[1-{\left(\frac{r-r_{in}}{s/2}\right)}^{m}\right]+m\left[\frac{Q}{A_{p}}-\frac{Q}{2\pi r_{\mathrm{int}}L}\right]\left(\frac{r-r_{in}}{s/2}\right)\left(\frac{r-r_{in}}{s/2}-1\right)\;;r_{in}\leq r\leq r_{\mathrm{int}}\left(=r_{in}+\frac{s}{2}\right)$$

(8) $$u_{r}^{\left(2\right)}\left(r\right)=\frac{Q}{2\pi rL}\;;r_{\mathrm{int}}\leq r\leq r_{out}$$

Here, the total perforated surface area of the pipe was indicated by $A_{p}\left(=\pi n_{c}n_{z}r_{p}^{2}\right)$. Equation and (7) is a power function where m-factor is denoted with m. It is a dimensionless number and is a function of the perforations scheme applied on the injection pipe and the geometric specifications of the tailings block, as shown in equation (9).

(9) $$m=\int \left(\beta ,R^{\prime }\right)$$

**Fig. 4:**
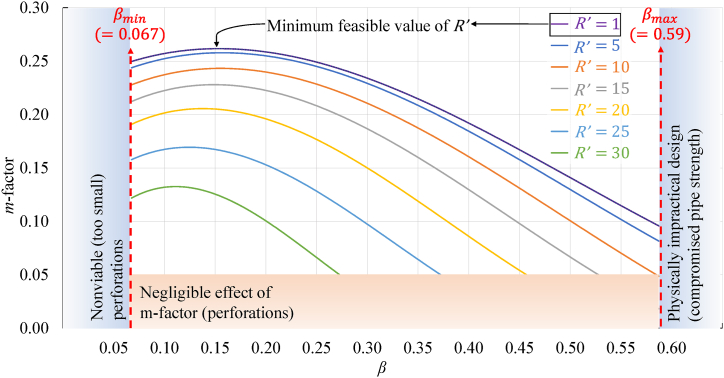
Variability in m-factors across distinct heights of tailings blocks and various patterns of perforations in the injection pipe (modified from Ref. [12]).

Here, β is a dimensionless number referring to the ratio of the total area of the perforations on the injection pipe surface ($A_{p})$ to the surface area of the enclosing cylindrical boundary encompassing the transition zone ($2\pi r_{\mathrm{int}}L)$. $R^{\prime }$ (shown in Fig. 2) indicates the ratio between the height of the block (h) and the size of the injection pipe diameter. Based on β and $R^{\prime }$, the value of m was approximated from the map given in Fig. 4 for using in equation (7).

Afterward, equations (7), (8) were employed separately in equation (2) and integrated with their designated range of r. This exercise resulted in the pressure profile through the mid-perforation for the zone 1 ($p_{{zone}_{1}}^{\prime }$) where the radial flow was activated. Adding the linear pressure drop in the zone 2 ($p_{{zone}_{2}}^{\prime }$) with $p_{{zone}_{1}}^{\prime }$ produced the total mid-perforation pressure profile ($p^{\prime }$), as demonstrated in equations (10), (11), (12).

(10) $$p_{{zone}_{1}}^{\prime }=\int_{r_{in}}^{r_{\mathrm{int}}}u_{r}^{\left(1\right)}\left(r\right)+\int_{r_{\mathrm{int}}}^{r_{out}}u_{r}^{\left(2\right)}\left(r\right)$$

(11) $$p_{{zone}_{2}}^{\prime }=Q\times \mu_{exh}\times \frac{\left\{1+\frac{1}{R_{wh}}\left(1-\frac{\pi }{4}\right)\right\}w-h}{4k_{t}hL}$$

(12) $$p^{\prime }=p_{{zone}_{1}}^{\prime }+p_{{zone}_{2}}^{\prime }$$

Here, $R_{wh}$ represents the ratio of the width (w) of the tailings block to its height (h). Equation (11) also includes the dark grey shaded area of the block (shown in Fig. 2) in the calculation, which was not included in the cylindrical flow assumption for zone 1. This was included by making necessary modifications to Darcy's law for linear flow. With the higher values of $R_{wh}$, the impact of this zone on the total pressure profile will become insignificant. On the other hand, the exhaust flow regimes within the porous media can be categorized based on the Reynolds number as follows: laminar flow (${Re}_{k}<1$), transitional flow ($1{<Re}_{k}<10$), and turbulent flow (${Re}_{k}>10$) [27]. As the operating configurations examined here were in the laminar flow regime, the amount of outflow in each section was calculated based on Darcy's law for radial coordinates [30] using the following equation.

(13) $$\Delta Q_{i}=\frac{\left(p_{g_{i}}-{{\Delta p}_{d}}_{i}/2\right)s}{\alpha }$$

In this context, the parameter α is contingent upon the specific arrangement of perforations employed in the injection pipe, as well as its influence on the pressure distribution along the pipe. Equations (14), (15) can be utilized to ascertain the value of α, catering to scenarios where the effect of the perforation arrangement holds significance or can be disregarded (pressure-optimized cases) correspondingly.

(14) $$\alpha =\frac{Lp^{\prime }}{Q}$$

(15) $$\alpha =\frac{\mu_{exh}}{2k_{t}}\left[\frac{1}{\pi }\ln\left(\frac{h}{d_{h}}\right)+\frac{\left\{1+\frac{1}{R_{wh}}\left(1-\frac{\pi }{4}\right)\right\}w}{2h}-0.5\right]$$

The gauge pressure, $p_{g_{i}}$, mentioned in equation (13) was iterated, using the (1 + 1) ROM in every i ^th^ section with the following equation. This is the combined pressure drop of TPL and IPL (injection pressure loss) for simultaneously occurring in Transport-2, and 3, respectively, as shown in equation (16).

(16) $$p_{g_{i+1}}={\Delta p}_{d_{i}}+{\Delta p}_{r_{i}}$$

Owing to the momentum dissipation resulting from the outflow through the perforations, an increase in pressure (recovery) spanning from one end of the perforation to the other was observed. This phenomenon arises because the outflow consists of a fluid with lower energy levels from the boundary layer in contrast to the fluid flow at the centerline of the pipe, leading to a simultaneous pressure rise in conjunction with the frictional influence [31]. This increase is expressed as pressure recovery, ${\Delta p}_{r_{i}}$ and equations (17), (18) (based on [32,33]) were applied iteratively to compute it.

(17) $${\Delta p}_{r_{i}}=k_{i}^{\prime }\rho_{exh}\left(v_{i}^{2}-v_{i+1}^{2}\right)$$

(18) $$k_{i}^{\prime }=\xi +\chi \left(\frac{v_{i}^{2}-v_{i+1}^{2}}{v_{i}^{2}}\right)$$

Here, the exhaust velocity inside the pipe and the momentum recovery in every i ^th^ section are denoted by $v_{i}$ and $k_{i}^{\prime }$. The coefficients ξ and χ are intricately linked with the ratio between the length and diameter of the pipe, undergoing iterative determination for each individual section [33]. Finally, the flow of exhaust progressed through the pipe in accordance with equation (19).

(19) $$Q_{i+1}=Q_{i}-{\Delta Q}_{i}$$

### 2.3 Financial analysis

The primary focus of the financial analysis of the concept was estimating operating costs. The installation of the necessary air handling units and construction of the piping for Transport-1 were regarded as capital expenditures. These were kept out of discussion in this investigation as these will be a one-time manageable capital investment. The economics of the entire operations can be evaluated by understanding and controlling the operating expenses involved. So, the target became to estimate the OPEX to sequestrate 1tonne of $CO_{2}$ in every operating year. The major costs contributing to the OPEX were the cost of piping, electricity, and materials handling. The materials handling cost indicated the arrangement of dry stack tailings bed and preparing it for diesel exhaust injection. However, as the dry stack tailings will have long-term operational benefits in tailings management cost, the associated expenditure related to materials handling was decided to exclude from the current investigation. This makes the total operating expenditure to capture 1tonne of $CO_{2}$ in every operating year (${ox}_{t}$) in mine tailings solely a function of the associated operating cost of piping (${ox}_{piping}$), and the electricity cost (${ox}_{elec}$), as shown in equation (20).

(20) $${ox}_{t}={ox}_{piping}+{ox}_{elec}$$

As previously mentioned, the total pressure drop (Δp) that the installed air handling units must counteract will equal the sum of the pressure differentials experienced across Transport-1, 2, and 3. The pressure drop in Transport-1 was estimated by altering equation (3). The (1 + 1)D ROM provided with the pressure drop associated in Transport-2, and 3. Based on Δp, and the total flow rate of the exhaust injection, the air handling unit could be chosen. Also, an additional 5% pressure drop was included in the calculations as a contingency. The energy consumed by the fan ($W_{fan})$ and the compressor ($W_{comp})$ in the operations was estimated by using equations (21), (22), respectively.

(21) $$W_{fan}=\frac{\left(\Delta p\right)Q_{t}}{\eta_{fan}}$$

(22) $$W_{comp}=\frac{{\rho_{exh}\times Q}_{t}{\times Cp}_{exh}}{\eta_{comp}}\left\{{\left(1+\frac{\Delta p}{p_{atm}}\right)}^{\left(1-\frac{1}{\gamma }\right)}-1\right\}T$$

where, $\eta_{fan}$ and $\eta_{comp}$ represent the efficiency of the fan and the compressor, respectively. $Q_{t}$ is the total volumetric flow rate and ${Cp}_{exh}$ is the specific heat of the exhaust, respectively. $p_{atm}$, γ, and and T indicate the atmospheric pressure, adiabatic index, and the temperature of the exhaust before it enters the compressor, respectively. As the operation in the discussion here was found dealings with higher pressure drops, especially with the tailings with low permeability, all the investigations here were carried forward with compressors as the air handling unit. The thermal characteristics of the exhaust were derived from the temperature-dependent thermophysical properties of air. This methodology aligns with the established practices observed in comparable studies focusing on the versatile utilization of diesel exhaust [25,34,35]. The available quantity of $CO_{2}$ in the diesel exhaust was estimated from the exhaust flow rate of the diesel generators mentioned in the provided datasheet from the manufacturers, assuming a 10% volumetric concentration of $CO_{2}$ [7]. Using equation (23), ${ox}_{elec}$ was evaluated in every scenario investigated where ${uc}_{elec}$ denotes the cost of electricity per kWh, and $M_{{CO}_{2}}$ represents the amount (in tonnes) of $CO_{2}$ sequestrated.

(23) $${ox}_{elec}=\frac{W_{comp}\times 365\times 24\times {uc}_{elec}}{M_{{CO}_{2}}}$$

The required energy to operate the air handling unit for flue gas injection in mine tailings to capture $CO_{2}$ needed to be delivered from the diesel-based power plant running in the mine site. So, it was necessary to keep a check on the energy requirement for running the operation throughout the study. To do so, a term noted as energy ratio ($R_{Ε}$) was introduced. It signifies the percentage ratio of the power needed for the sequestration operation to the sequestrated power.

The entire dry stack tailings bed was conceptualized to be divided into identical blocks with the same h, l, and w. The effective execution of the envisioned concept hinges upon a uniform outflow of exhaust through the perforations into the tailings, spanning the entirety of the length of the embedded injection pipe. In the cases of high permeable tailings, where IPL was significantly lower than TPL, the injection of the gas into the tailings became easier than transporting inside the pipe. Such scenario resulted in the major portion of the gas injection in the initial segment of the block. The smoothness of the outflow distribution could be determined by the flow uniformity index ($f_{ui}$), as shown in equation (24).

(24) $$f_{ui}=\frac{\left(\Delta Q_{1}\right)L}{Q_{t}s}$$

where, $\Delta Q_{1}$ indicates the outflow from the 1st discretized section of the pipe. In an ideal smooth distribution scenario, $f_{ui}$ will be equal to 1. In case of an abrupt distribution of the outflow ($f_{ui}>1$), a substantial portion of the tailings will not be sequestrated because of the exhaust failing to reach them. To avoid this mishap, the dimensions of the injection pipe, to be situated at the center of each block, were subsequently established by evaluating the ratio between TPL and IPL. Having injection pipes with larger diameters resulted in less frictional losses advocating a lower value in TPL and a brought the necessary balance between TPL and IPL. In all the investigations conducted here, this exercise was followed thoroughly to find the optimum diameter for the injection pipe. It was observed that an acceptable smooth distribution of the outflow was present when IPL was at least 15 times higher than TPL. In general, the low permeability of the tailings required the installation of injection pipes with larger diameters.

The number of perforation pipes ($n_{pipe}$) was dependent on the number of the tailings blocks employed in the operation, and they were equal. The total operating cost for injection piping was estimated based on the total length of piping required ($n_{pipe}L$) and the cost of the piping per meter (${uc}_{piping}$). Using equation (25), ${ox}_{piping}$ was evaluated in every scenario investigated here.

(25) $${ox}_{piping}=\frac{n_{pipe}L\times {uc}_{piping}}{t_{piping}\times M_{{CO}_{2}}}$$

where, $t_{piping}$ refers to the re-piping period. As previously mentioned, after one layer of tailings is fully sequestrated, a new layer of tailings will be needed with a new set of injection pipes. The frequency of re-piping activity was measured with $t_{piping}$ (in years). If $t_{piping}$ was less than 1year, that meant that a new layer of tailings with new sets of injection pipes were needed to be installed even before completing 1year of operation. In such cases, the cost of piping in an operating year will be higher. This could be experienced when the tailings had a lower capacity to sequestrate $CO_{2}$ than the available or targeted amount to capture. On the other hand, tailings with a higher reactivity potential could have operations where $t_{piping}$ was more than 1year. However, this was an operating parameter and could be controlled by appropriately setting up the operation. It is pertinent to note that the term "operating year" utilized in this study does not invariably denote a calendar year. The operating year indicates an operation span of 365 days where diesel exhaust is injected into the tailings for capturing $CO_{2}$. However, these 365 days do not necessarily need to be continuous. Considering the application of the present study in remote, off-grid locations, it is essential to mention that the operational continuity may not be sustained throughout the entire year due to harsh climatic conditions and other challenges. Therefore, the term "operating year" was introduced and consistently employed throughout the study to enhance investigations clarity.

As the carbon capture was guided by the reactivity potential of the tailings, it was important the inclusion of that in evaluating the financial aspects of the proposed concept here. A parameter called reactivity index (RI) was introduced and was computed as shown in equation (26).

(26) $$RI=\frac{M_{{CO}_{2}}}{M_{tailings}}$$

where, $M_{tailings}$ indicates the amount of the tailings (in tonnes) that was required to sequestrate $M_{{CO}_{2}}$. RI is a crucial parameter which is dependent on the geo-physical characteristics of tailings and some other operational conditions. The average power demand of the mine, and the amount of the available tailings along with RI governed $M_{tailings}$ by influencing the system design.

## 3 Results and discussion

### 3.1 Establishment of energy-optimum scenario for flue gas injection in tailings block

Before investigating the financial details of the proposed concept, it was essential to check the viability of the application of the extended (1 + 1)D ROM in estimating the pressure profile for perforated pipes embedded in rectangular tailings block. To evaluate the actuality of the (1 + 1)D ROM, four perforation designs ($n_{c}=4$, 6, 8, and 10) introduced in Ref. [12] were selected. For each perforation case, two different perforation ratios ($A_{r}=30\%$, and 50%) were picked. Two different heights were chosen for the tailings blocks, along with four different width-to-height ratios ($R_{wh}$). In total, sixty-four different configurations were prepared for perforated injection pipes installed in the tailings bed for the extended (1 + 1)D ROM. Simultaneously, the same configurations were replicated with the 3D FE model. The key parameters used in the investigations conducted in this section of the study are given in Table 1.

**Table 1:** Key parameters used in constructing the (1 + 1)D ROM and 3D FE models for results comparison.

Parameters	Value
Density of the tailings ($\rho_{tailings}$)	$2600\;\text{kg}/m^{3}$ [36]
Height of the tailings block (h)	2m
Length of the tailings block or the injection pipe	1.02m
Injection flow rate (Q)	$1.7\times 10^{-3}\;m^{3}/s$
Air volume fraction or porosity of the tailings ($\varepsilon_{t})$	35% [37]
Injection temperature	80° C
Permeability of the tailings ($K_{t})$	$4.8\times 10^{-11}\;m^{2}$

**Fig. 5:**
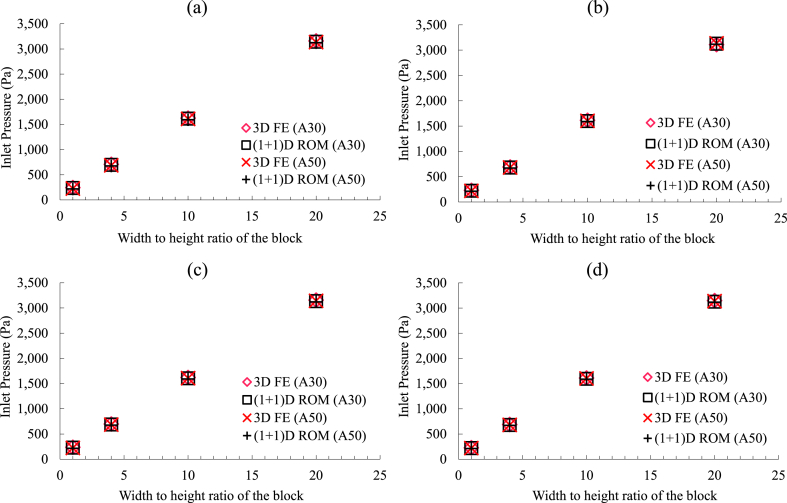
Comparison of the inlet pressures obtained from the 3D FE models and (1 + 1)D ROM for rectangular tailings block ($R^{\prime }=5$) for different perforation configurations based on the number of the perforations around the circumference of the pipe: (a) $n_{c}=4$, (a) $n_{c}=6$, (a) $n_{c}=8$, and (a) $n_{c}=10$.

**Fig. 6:**
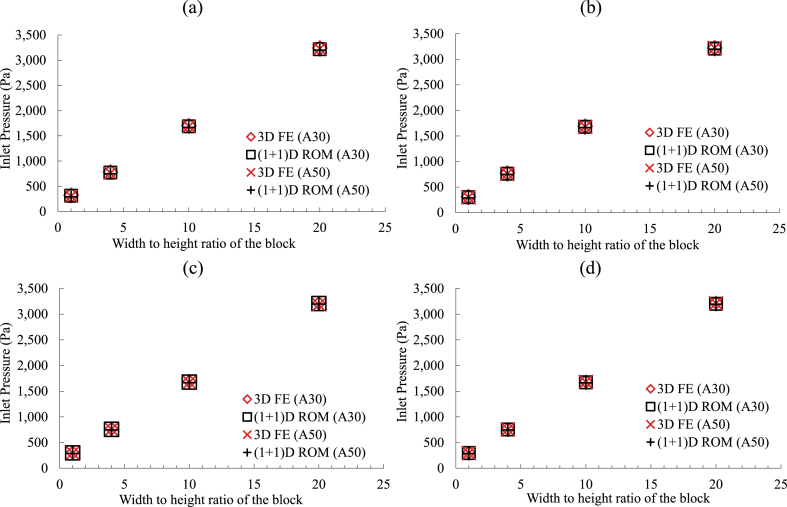
Comparison of the inlet pressures obtained from the 3F FE models and (1 + 1)D ROM for rectangular tailings block ($R^{\prime }=12$) for different perforation configurations based on the number of the perforations around the circumference of the pipe: (a) $n_{c}=4$, (a) $n_{c}=6$, (a) $n_{c}=8$, and (a) $n_{c}=10$.

The results for the inlet pressure obtained for all the sixty-four cases from the (1 + 1) ROM were compared with the outcomes from the 3D FE models. The comparisons for all these configurations are shown in Fig. 5(a–d), and Fig. 6(a–d), respectively for the tailings blocks that had $R^{\prime }$ of 5, and 12.

For all the cases tested here, the (1 + 1)D ROM and the 3D FE models were found to be in good agreement in predicting the inlet pressures of the perforated pipes embedded in the rectangular blocks of the tailings. The maximum difference between the results obtained was less than 3%, and it was observed for the smallest width-to-height ratios tested here. With the larger-sized blocks, and higher intensity of the perforations, the effect of perforations in the pressure profile became insignificant. The target of this section of the study was to prove that the injection of diesel exhaust in mine tailings for carbon sequestration can be designed in an optimal way for energy management. To achieve such a scenario, two factors were necessary to confirm. One of them was to ensure that the outflow through the perforations of the injection pipe was consistent into the tailings throughout the length of the tailings block. This could be ensured by sizing the perforation schemes previously established in Ref. [12]. The second one was to ensure that the pressure drop the system would need to overcome was mostly the pressure drop due to the injection in porous tailings. This can be achieved if the impact of the perforations on the pressure profile becomes insignificant. When the pressure drop is optimal, the required energy to overcome that will also be the same. This was previously shown as attainable in a cylindrical block [12]. Here, the investigations were continued to ensure the possibility of reaching such a setup in rectangular tailings blocks.

**Fig. 7:**
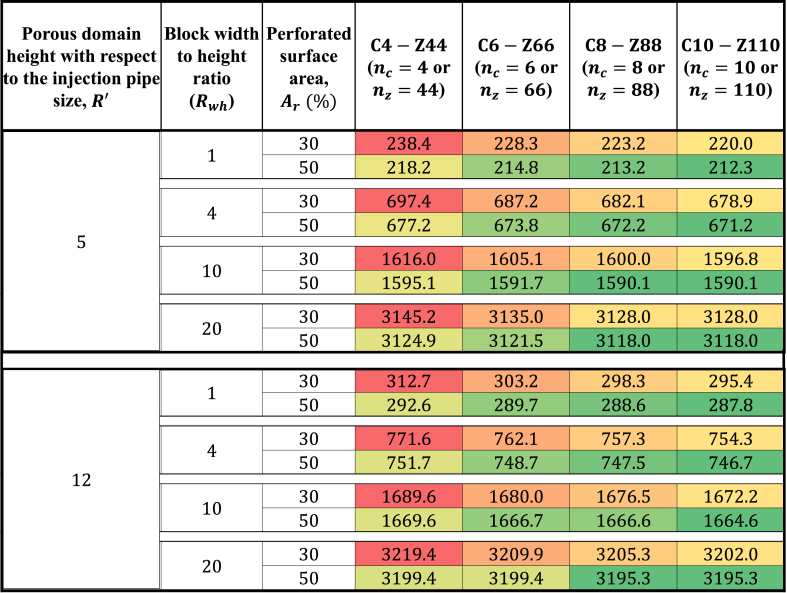
Heat maps illustrating variations in inlet pressure for tailings blocks of different dimensions and various perforation arrangements.. (Red and green color denote the maximum and the minimum values in the range, respectively.). (For interpretation of the references to color in this figure legend, the reader is referred to the Web version of this article.)

A combination of multiple heat maps is given in Fig. 7, showing the variations in the inlet pressures for different perforation schemes applied on the injection pipes installed in different dimensions of rectangular tailings block. For each block size, a heat map is curated, demonstrating the effect of different perforation schemes on the inlet pressure. These maps can be applied in the decision-making process for choosing a perforating scheme for the system design. For example, when the tailings block had 5-times height of the injection pipe diameter along with the $R_{wh}$ of 1 (square-shaped block), the least-dense perforation scheme (4 perforations around the circumference of the pipe with 30% of the pipe surface perforated) applied here resulted in an inlet pressure drop of 238.4Pa. This could be reduced by 8.5% by making 50% of the pipe surface perforated. It can be further reduced to 214.8Pa by increasing the number of perforations around the circumference of the pipe by 2. Further densifying the perforation scheme did not bring any significant reduction in the inlet pressure estimates. From the heat maps, it can also be understood that the larger block sizes required a less dense perforation scheme to achieve the pressure-optimum scenario. For example, blocks with $R^{\prime }=12$, which had a $R_{wh}$ of 4, the difference between the inlet pressure obtained with the least and most dense perforation arrangement evaluated here was 3.3%. That means that using the least dense perforation scheme could result in an additional 3.3% pressure drop in comparison to the pressure-optimum case. However, this value decreased to 0.7% when the $R_{wh}$ became 20. Such analyses showed that it was possible to achieve an energy-optimum design for flue gas injection in mine tailings for carbon capture with the extended (1 + 1)D ROM. Further calculations on the financial aspects of the proposed concept were carried out with presuming an energy-optimum design applied.

### 3.2 Financial analysis

As mentioned earlier, in the proposed concept, the injection of diesel exhaust will start with one-layer dry stack tailings where the initial set of injection pipes will be embedded. After the complete reaction of one layer of tailings, a fresh layer can be established by employing a new set of injection pipes. This process was called the re-piping in this study, and the time difference between the piping and the re-piping process was noted as re-piping period. Two hypothetical scenarios were constructed, based on the duration of the re-piping periods. In the first one, a fixed re-piping period of 1 year was considered. For the second one, the re-piping period was kept variable. For each scenario, the operating expenditure was evaluated for a range of permeabilities ($K_{t})$ and reactivity indexes (RI) of the tailings along with the dimensions of the tailings blocks. The primary parameters used in the investigations conducted in this section of the study are given in Table 2, unless mentioned otherwise.

**Table 2:** Base parameters used in the financial aspects analysis.

Parameters	Value
Density of the tailings ($\rho_{tailings}$)	$2600\;\text{kg}/m^{3}$ [36]
Height of the tailings block (h)	2m
Length of the tailings block or the injection pipe	1000m
Transport-1 distance	2000m
Air volume fraction or porosity of the tailings ($\varepsilon_{t})$	35% [37]
Injection temperature of the exhaust	80° C
Number of perforations around the circumference of the pipe ($n_{c})$	10
Perforated surface area of the injection pipe ($A_{r})$	50%
Efficiency of the air handling equipment	65%
Cost of electricity $\left({uc}_{elec}\right)$	0.26C$/kWh
Volumetric percentage of $CO_{2}$ in diesel exhaust	10%

The cost for piping (${uc}_{piping}=67\;C\$/m$) was established based on data obtained from a reputable manufacturer for a specific size (16inches diameter) of a pipe. To account for the variations in pipe diameters used in this study, a functional relationship between the diameter of the pipe and its corresponding cost was developed. This approach ensured that the cost estimation remained accurate and adaptable to different pipe sizes as needed throughout the study. By utilizing the manufacturer's data and incorporating a scalable cost model, a realistic representation of expenses associated with piping in this research was maintained, promoting transparency and accuracy in our economic evaluations.

#### 3.2.1 Fixed re-piping period approach

**Fig. 8:**
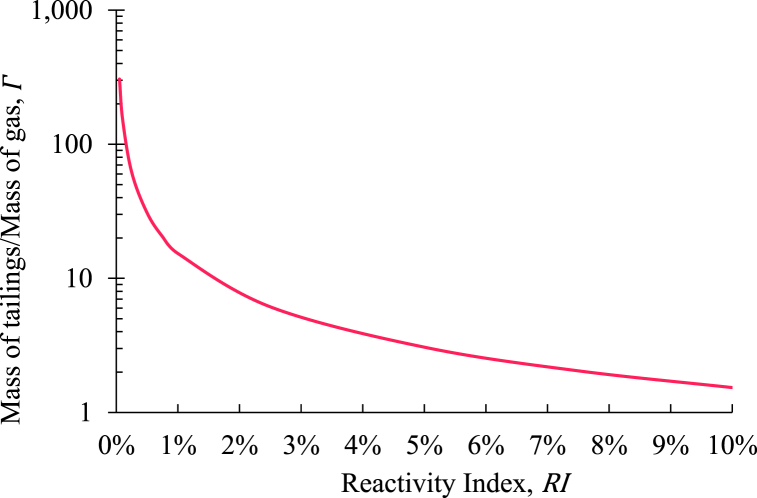
Relationship between the reactivity indexes of the tailings and the ratio of the mass of the tailings to the mass of the exhaust injection.

At first, based on RI, a relationship between the amount of the tailings and the amount of the exhaust being injected into that tailing was established. To sequester the same amount of power, depending on the RI of the tailings, different amounts of tailings will be required. Higher RI of the tailings will advocate for higher rate carbon sequestration potential, which means that it will require less amount of tailings to sequestrate the same amount of power in comparison to the tailings with lower values of RI. Considering the injection temperature of 80°C, the mass flow rate of diesel exhaust injection was estimated for 1MW of power sequestration. For an operating span of 1year, the amount of the tailings required for sequestrating 1MW of power was evaluated using RI. That exact amount of tailings was considered available and utilized in 1year, and using that, the ratio of the mass of the tailings to the mass of the exhaust being injected into that was expressed as Γ in Fig. 8. The relationship between Γ and RI exhibited a direct proportionality.

After understanding the significance of Γ, the ${ox}_{piping}$ for sequestrating 1MW of power in mine tailings with different values of RI and $K_{t}$ was decided to be evaluated. A comprehensive range for RI ranging from 0.1% to 5% was selected, along with a possible range for $K_{t}$ for the mine tailings. The amount of the tailings utilized here was directly determined from the associated values of RI. That means that with the lower values of RI, the same amount of exhaust gas was injected in a higher volume of tailings compared to the tailings with the higher values of RI. For example, for RI of 0.5% (Γ=30.6), 1.7 million tonnes (MT) of tailings will be required over the span of 1year of operating period to sequestrate 1MW of power (equivalent to 8.6MT of $CO_{2}$). This will reduce to 0.3MT for RI of 2.5% (Γ=6.1). However, for both cases, the same amount of diesel exhaust will be injected, and the $M_{{CO}_{2}}$ will be the same.

**Fig. 9:**
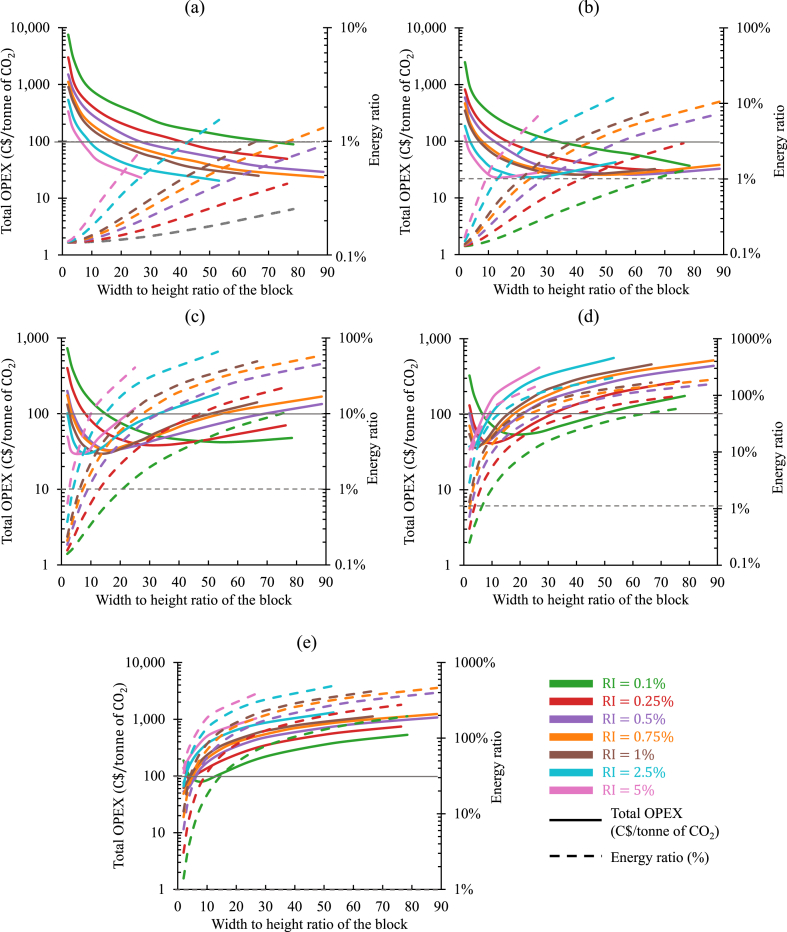
Total operating expenditure and the associated energy ratios per tonne of $CO_{2}$ captured targeting 1MW power sequestered in the reactivity-index driven amount of mine tailings for different permeabilities at fixed re-piping period scenarios: (a) $K_{t}=10^{-10}{\;m}^{2}$, (b) $K_{t}=10^{-11}{\;m}^{2}$, (c) $K_{t}=10^{-12}{\;m}^{2}$, (d) $K_{t}=10^{-13}{\;m}^{2}$, and (e) $K_{t}=10^{-14}{\;m}^{2}$. (Operating expenditures (solid lines, left axis) and energy ratios (dashed lines, right axis) depicted for varying reactivity indexes of the tailings, distinguished by different colors.). (For interpretation of the references to color in this figure legend, the reader is referred to the Web version of this article.)

After having an estimate for the quantity of tailings to be employed ($M_{tailings}$) for each value of RI, attention was paid to sizing the tailings blocks. Keeping the same height for all the blocks, the width was made variable. Based on $M_{tailings}$ and $R_{wh}$, the number of blocks (or perforated injection pipes) needed was determined. For all cases investigated here, $t_{piping}$ was presumed to be 1year, meaning that the $M_{tailings}$ determined were considered available and prepared for the injection prior to the initialization of the operation. Then the injection of flue gas was continued for 1year. The ${ox}_{t}$ for each scenario was evaluated and then shown in Fig. 9(a–e). The results are categorized by the values of $K_{t}$, and ${ox}_{t}$ (on the left axis) along with their associated $R_{Ε}$ (on the right axis) values presented together. Thresholds of 100C$ (indicated with the solid black line) and 1% (indicated with the dashed black line) were set respectively for ${ox}_{t}$ and $R_{Ε}$ for performance evaluation of the proposed design concepts.

For higher values of $K_{t}$, many cases fell under the threshold for both ${ox}_{t}$ and $R_{Ε}$. With the lower values for $K_{t}$, though there were some cases under the threshold value for ${ox}_{t}$, almost no case remained under the threshold value that was set for $R_{Ε}$. For example, for $K_{t}=2\times 10^{-10}{\;m}^{2}$, only 3 cases had $R_{Ε}$ above 1%. Moving to $K_{t}=10^{-13}{\;m}^{2}$, there were only 3 cases that had $R_{Ε}$ below 1% for all the values of RI considered. It happened as the system needed to overcome a huge IPL when the tailings had lower permeabilities. This necessitated a significant amount of energy consumption by the installed air handling units to counteract the induced pressure losses. For the same reason, the minimum value for ${ox}_{t}$ was observed with the higher values of $R_{wh}$ for cases with higher values of $K_{t}$. For example, in Fig. 9(b) ($K_{t}=10^{-11}{\;m}^{2}$), for RI=0.75%, the minimum ${ox}_{t}$ was observed with the design when tailings blocks had a $R_{wh}$ of 44. This decreased to 4, in Fig. 9(d) ($K_{t}=10^{-13}{\;m}^{2}$), for the same reactivity index of the tailings. As mentioned earlier, ${ox}_{t}$ had only two active components: operating expenditure for piping and electricity. When the permeability of the tailings is high, the operating expenditure per tonne of CO_2_ sequestrated was dominated mainly by the ${ox}_{piping}$. In such cases, as IPL was very low, going with a higher $R_{wh}$ resulted in the lowest ${ox}_{t}$. However, with the decrease in $K_{t}$, IPL increased, and the ${ox}_{elec}$ became dominant in the ${ox}_{t}$. This resulted in the observation of the minimum ${ox}_{t}$ in the cases that had lower values of $R_{wh}$ for low permeable tailings.

**Fig. 10:**
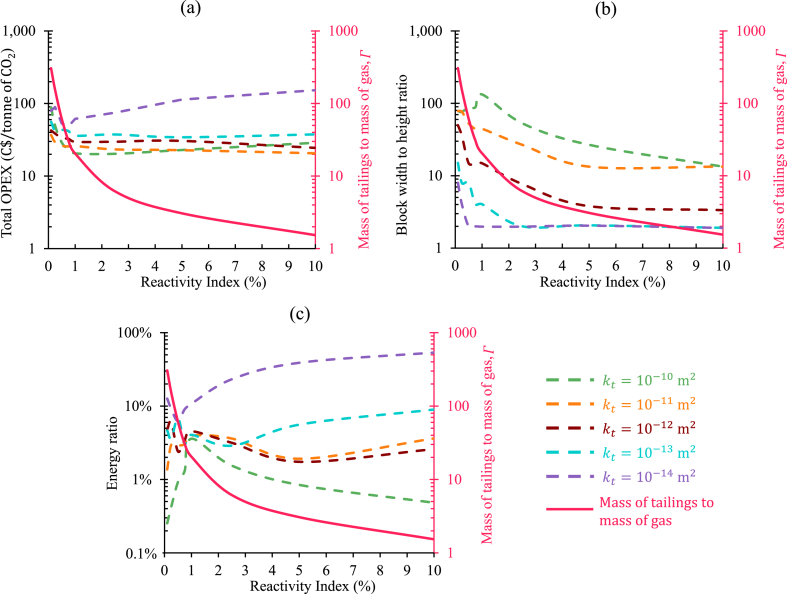
Correlation analysis elucidating the interdependence of reactivity indexes of the tailings and the mass ratio between tailings and exhaust injection in (a) the minimum OPEX for each reactivity cases displayed in Fig. 8, (b) the associated width to height ratio of the block size for the minimum OPEX cases, and (c) the associated energy ratio for each of the minimum OPEX cases. (Visualization of the mass ratio between tailings and exhaust injection, shown as solid red lines on the right axis of each figure, alongside minimum OPEX, width to height ratio of the block, and energy ratio (depicted by dashed lines, on the left axis) within the designated graph. Different colors denote distinct tailings permeabilities.). (For interpretation of the references to color in this figure legend, the reader is referred to the Web version of this article.)

Identifying the optimal point between ${ox}_{elec}$ and ${ox}_{piping}$ was vital in the cost estimation for ${ox}_{t}$. The optimal point did not necessarily have the lowest value for either ${ox}_{elec}$ or ${ox}_{piping}$. However, the combination of them generated the minimum ${ox}_{t}$. The minimum operating expenditure to sequestrate 1tonne of $CO_{2}$ in every operating year for each case, presented in Fig. 9, was identified. Fig. 10(a) shows these minimum ${ox}_{t}$ (on the left axis) values gathered and their designated Γ (on the right axis) with respect to RI. The associated $R_{wh}$ and $R_{Ε}$ for these minimum ${ox}_{t}$ cases are shown on the left axis in Fig. 10(b) and (c), respectively. The results were categorized based on the permeability of the tailings.

In Fig. 10(a), all but 2 cases had an ${ox}_{t}$ below 100C$. In almost all cases, the higher permeability configurations resulted in lower ${ox}_{t}$. The cases with RI of 0.75% can be discussed as examples here. For $K_{t}=10^{-14}{\;m}^{2}$, the minimum operating expenditure to sequestrate 1tonne of $CO_{2}$ in every operating year was 48C$. This dropped to 26C$ when $K_{t}$ was $10^{-11}{\;m}^{2}$. Though almost all the configurations had an ${ox}_{t}$ that was lower than the threshold value set, the majority of these were above the individual threshold set for $R_{Ε}$. As shown in Fig. 10(c), only a few configurations that had the highest permeability tested here had a $R_{Ε}$ below 1%. This was also reflected in Fig. 10(b), where the $R_{wh}$ associated with the minimum ${ox}_{t}$, in most cases, was less than 11. While the $R_{wh}$ was low, the ${ox}_{t}$ was dominated by the ${ox}_{piping}$, as IPL in such cases was comparatively lower. So, the energy needed to operate the air handling units was also on the lower side. However, with the increase of $R_{wh}$, pushing the gas in the lateral direction became difficult, and this made the ${ox}_{elec}$ the most contributing to the ${ox}_{t}$. That is why the highest permeability cases had the minimum ${ox}_{t}$ observed where they had comparatively higher $R_{wh}$. In these cases, the operating expenditures were mostly dominated by the ${ox}_{piping}$. Because the high permeable tailings required injection pipes with larger diameters to make TPL reasonably lower than IPL for a smooth outflow distribution. However, it is also important to mention that pushing the exhaust in the lateral direction for such a long distance could be challenging to execute. A holistic approach is crucial to find out the optimal point for all these factors.

So far, in the financial analysis, the amount of the tailings utilized for carbon sequestration was guided by Γ. This generalized approach was a good strategy to initiate the discussion around the financials of carbon sequestration operation in mine tailings through the injection of diesel exhaust. However, restricting $M_{tailings}$ based on Γ, resulted in some complications in the results shown in Fig. 9, Fig. 10. For lower values of $K_{t}$, the higher values of RI cases had higher ${ox}_{t}$ than those with lower RI values in multiple configurations. The design configurations, where $K_{t}=10^{-13}{\;m}^{2}$ can be discussed as examples. For RI of 0.25%, the ${ox}_{t}$ was 42C$, when $R_{wh}$ was 8. For the exact dimension of the tailings blocks, the ${ox}_{t}$ increased to 74C$ when the reactivity index became 10 times higher (RI=2.5%). The reactivity of the tailings is the most crucial factor for the successful large-scale implementation of carbon sequestration in mine tailings through flue gas injection. The higher the reactivity of the tailings, the lower the ${ox}_{t}$ should be. The reason behind such misleading results was the constraint set for $M_{tailings}$ by Γ. This resulted in an equal amount of gas injection across varying quantities of tailings. When the reactivity was lower, a larger amount of tailings was required. Conversely, as RI increased, the $M_{tailings}$ decreased. The injection of the same amount of flue gas (as all cases targeted to sequestrate 1MW of power) resulted in tremendously higher IPLs, when the $M_{tailings}$ was lower (in higher values of RI) because of higher injection flux. Interpretations of such results could be deceptive, and to avoid that, the constraints set for the financial analysis was shaped to represent the results differently.

**Fig. 11:**
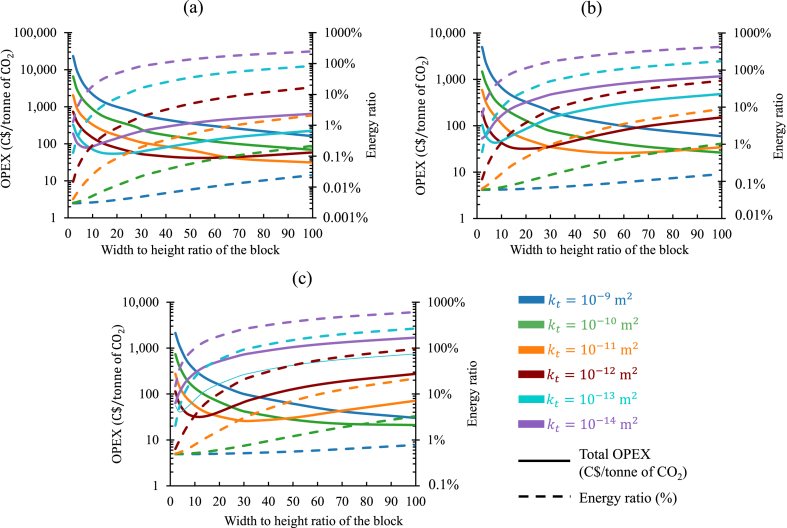
Total operating expenditure per tonne of $CO_{2}$ captured and the associated energy ratio for sequestrating (a) 0.5MW, (b) 2.7MW, and (c) 8.05MW of power in 4.5MT of tailings with reactivity index of 0.1%, 0.5%, and 1.5%, respectively, in a fixed re-piping period scenario. (Operating expenditures (solid lines, left axis) and energy ratios (dashed lines, right axis) are depicted for varying permeabilities of the tailings, distinguished by different colors in each figure.). (For interpretation of the references to color in this figure legend, the reader is referred to the Web version of this article.)

In a practical scenario, a mining operation will always have a certain quantity of tailings available annually, along with a specific power demand for operations. Both will vary from mines to mines and depend on several factors, e.g., mining method, ore type, processing technology, operating practices, and so forth. Therefore, in the real-life implementation of the proposed concept, the reactivity index of the tailings will be first estimated. After having a comprehensive understanding of the reactivity potential, based on the quantity of tailings available from the operation, a certain percentage of power will be targeted to sequestrate in a specific operating period. To further explain the approach, the necessary parameters were adapted for a mine operating at a remote, off-grid location in the Northwest Territories of Canada from Ref. [38]. This mine had an average power demand of 8.05MW, and around 4.5MT of tailings was available yearly. Three different values for RI were selected, and they were 0.1%, 0.5%, and 1.5%. Based on the methodology established in this study, it was evaluated that employing 4.5MT of tailings in an operating period spanning 1year, 0.5MW, 2.7MW, and 8.05MW of power can be sequestrated, respectively, with the chosen values for RI. The operating expenses (depicted with the solid lines on the left axis) and the energy ratio (depicted with the dashed lines on the right axis) for each scenario are shown in Fig. 11(a–c) for a range of permeabilities.

For all the cases shown here, the minimum ${ox}_{t}$ was consistently observed in the middle of the permeability range tested here, regardless of the reactivity index. Based on the analysis, the most suitable operating point could be found between $K_{t}$ of $10^{-11}{\;m}^{2}$ and $10^{-12}{\;m}^{2}$, in terms of permeability. In that range, the power consumption by the air handling units were found to be acceptable and having a good ratio between TPL and IPL allowed to use a reasonable injection pipe size. Before that (for higher permeabilities), the ${ox}_{t}$ was primarily dominated by the ${ox}_{piping}$ because of the required installation of the larger-sized injection pipes. Afterward (for lower permeabilities), the ${ox}_{elec}$ became more impacting, which resulted in an increase in the ${ox}_{t}$.

For all the configurations tested here, in the higher end of the permeability range ($10^{-9}{\;m}^{2}$ and $10^{-10}{\;m}^{2}$), the lowest ${ox}_{t}$ was achieved with the largest block sizes because of the very low IPL occurred in such cases. For example, for $K_{t}=10^{-10}{\;m}^{2}$, the minimum ${ox}_{t}$ was witnessed with a $R_{wh}$ of 99 for all reactivity index cases tested here. With a decrease in permeability afterward, the $R_{wh}$ associated with the minimum ${ox}_{t}$ dropped, except for RI=0.1% cases, as the injection flow rate was still significantly low in this configuration. For $K_{t}=10^{-12}{\;m}^{2}$, the minimum ${ox}_{t}$ could be achieved with a $R_{wh}$ of 15 while targeting to sequestrate 2.7MW (RI=0.5%), and 8.05MW (RI=1.5%) of power. In these two cases of RI, for the lowest permeability tested here ($K_{t}=10^{-14}{\;m}^{2}$), the minimum ${ox}_{t}$ was achieved with the lowest value of $R_{wh}$ (=2).

The sequestration of 0.5MW, 2.7MW, and 8.05MW of power would result in the decarbonization of approximately 6.2%, 33.5%, and 100% of the average electricity generation of the mine. So, it was important to check the energy ratios for the cases considered here. For $K_{t}=10^{-9}{\;m}^{2}$, all the minimum ${ox}_{t}$ cases had a $R_{Ε}$ below 1% for all the values of RI investigated here. The reduction in the permeability afterward resulted in the increased standings of $R_{Ε}$. For example, for $K_{t}=10^{-11}{\;m}^{2}$, the minimum ${ox}_{t}$ cases had a $R_{Ε}$ of 2%, 2%, and 4%, respectively, for RI of 0.1%, 0.5%, and 1.5%. In some cases, due to the lower permeability of the tailings, the $R_{Ε}$ could be significantly higher than the previously set threshold of 1%. For example, the minimum ${ox}_{t}$ case for $K_{t}=10^{-14}{\;m}^{2}$, had a $R_{Ε}$ of 6% and 15%, respectively, for RI of 0.5%, and 1.5%. However, it is also important to take into consideration that these cases targeted to decarbonize the one-third and the entire average power generation of the mine, correspondingly. When choosing an optimal design zone for operating, such factors should also be included in decision-making process.

In this representation of the cost analysis (shown in Fig. 11), different amounts of $CO_{2}$ (different injection rates) were sequestrated in the same amount of tailings based on their RI. On the other hand, in the previous representations (shown in Fig. 9, Fig. 10) the same amount of $CO_{2}$ (same injection rate) was captured in different amounts of tailings, guided by the associated values of RI. Both situations were governed by the ratio of the mass of the tailings to the mass of the exhaust injection (Γ). However, in the first one (Fig. 9), it was used to determine the amount of the tailings to be utilized, and in the later one (Fig. 11), it was utilized to estimate the exhaust injection rate. Both tactics have the potential to be adopted in the decision-making process for carbon capture in mine tailings through flue gas injection.

#### 3.2.2 Variable re-piping period approach

The analyses conducted thus far had a fixed re-piping period of 1year meaning a new layer of tailings with a new set of injection pipes would be installed after 1year of operation. So, the amount of tailings laid down at the beginning had enough potential to capture carbon for 1year of operation. While this scenario could be deemed ideal, it may not always be assured. In real-world applications, the power generation of a mine and the tailings available from the operation could lead to a re-piping period of more or less than 1year. If the tailings are highly reactive and the mine has a comparatively low power demand, the re-piping period is likely more than 1year of operation. Here, the cost of piping will be lower as it will be less frequent. On the other hand, if the reactive potential of the mine tailings is on the lower spectrum of RI, then the targeted power may require multiple layers of tailings installation, making the operating period less than 1year. In such cases, the piping cost will go higher as installation of a set of injection pipes will be required more frequently. In this hypothetical scenario, it was assumed to have access to additional stockpiles of tailings to support the multiple re-piping activity in the same operating period.

**Fig. 12:**
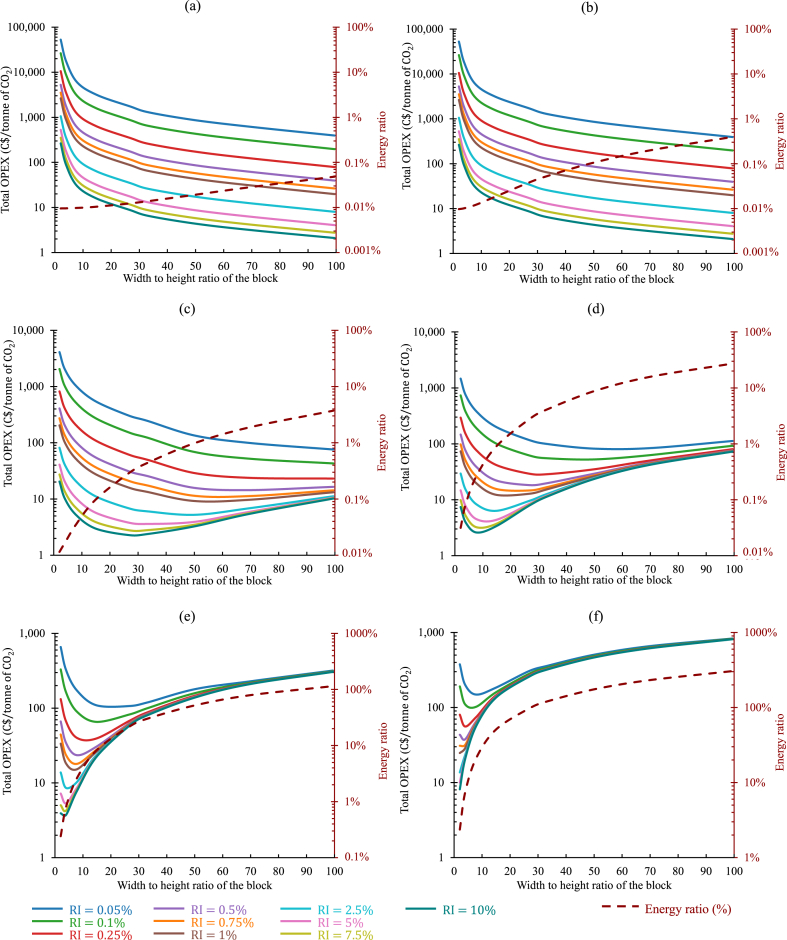
Comparison of the total operating expenditure per tonne of $CO_{2}$ captured and the corresponding energy ratio for the sequestration of 1MW power in 4.5MT of tailings, considering distinct permeabilities under a variable re-piping period scenario: (a) $K_{t}=10^{-9}{\;m}^{2}$, (b) $K_{t}=10^{-10}{\;m}^{2}$, (c) $K_{t}=10^{-11}{\;m}^{2}$, (d) $K_{t}=10^{-12}{\;m}^{2}$, (e) $K_{t}=10^{-13}{\;m}^{2}$, and (f) $K_{t}=10^{-14}{\;m}^{2}$. (Operating expenditures (solid lines, left axis) and energy ratios (dashed lines, right axis) depicted for varying reactivity indexes of the tailings, distinguished by different colors in each graph.). (For interpretation of the references to color in this figure legend, the reader is referred to the Web version of this article.)

**Fig. 13:**
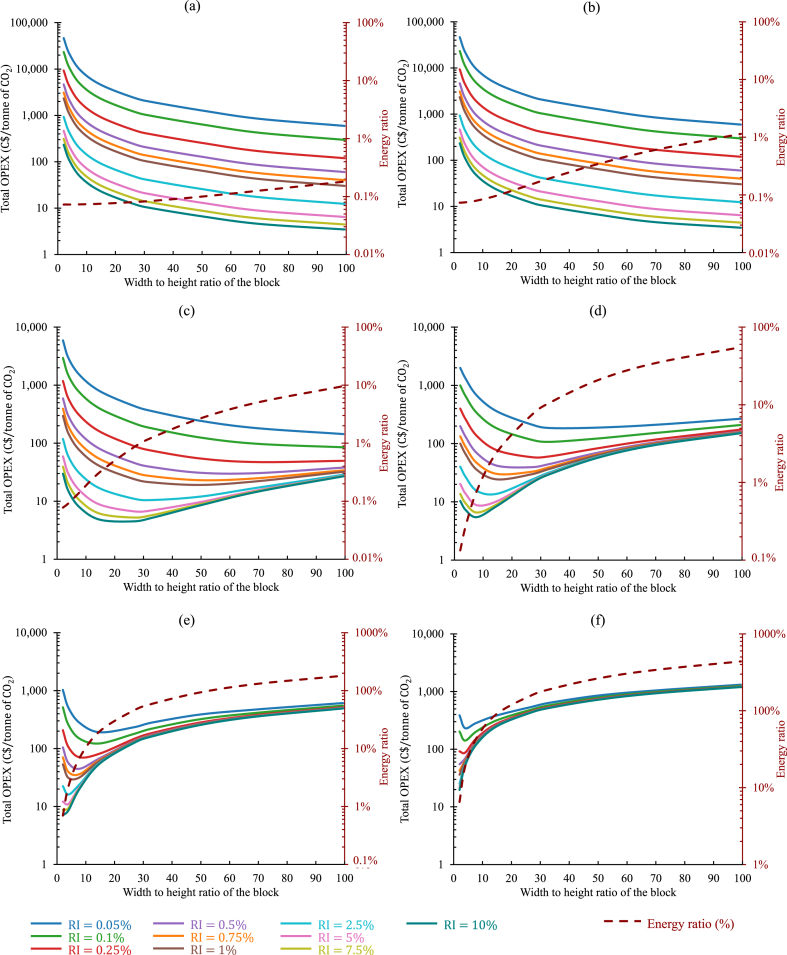
Comparison of the total operating expenditure per tonne of $CO_{2}$ captured and the corresponding energy ratio for the sequestration of 3MW power in 4.5MT of tailings, considering distinct permeabilities under a variable re-piping period scenario: (a) $K_{t}=10^{-9}{\;m}^{2}$, (b) $K_{t}=10^{-10}{\;m}^{2}$, (c) $K_{t}=10^{-11}{\;m}^{2}$, (d) $K_{t}=10^{-12}{\;m}^{2}$, (e) $K_{t}=10^{-13}{\;m}^{2}$, and (f) $K_{t}=10^{-14}{\;m}^{2}$. (Operating expenditures (solid lines, left axis) and energy ratios (dashed lines, right axis) depicted for varying reactivity indexes of the tailings, distinguished by different colors in each graph.). (For interpretation of the references to color in this figure legend, the reader is referred to the Web version of this article.)

**Fig. 14:**
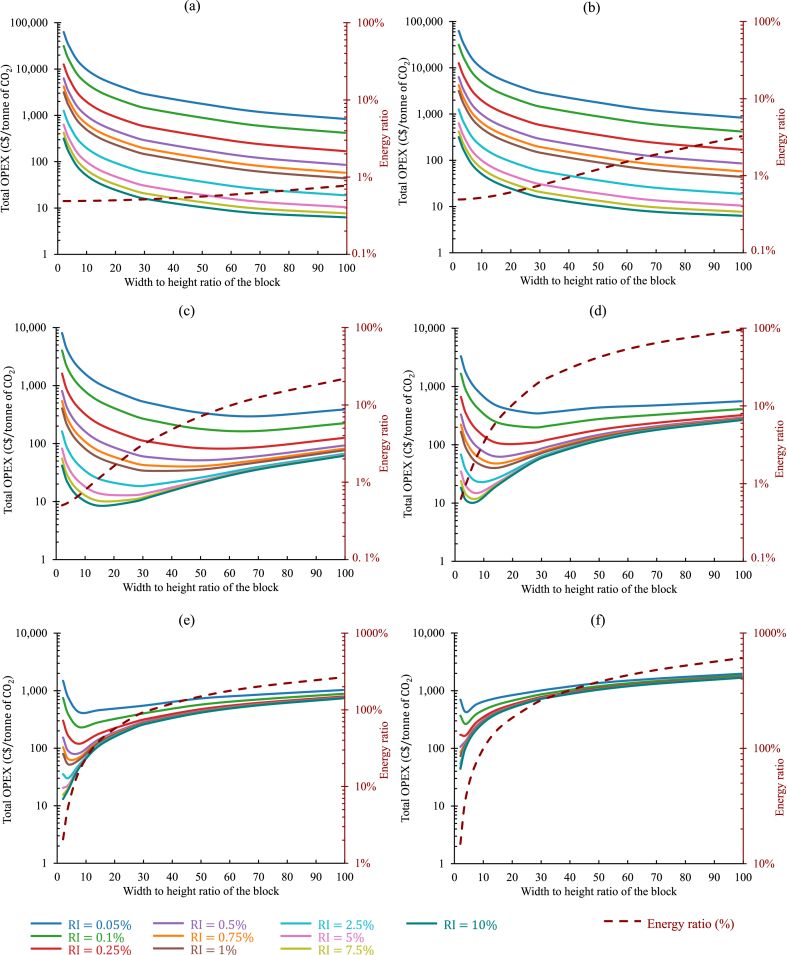
Comparison of the total operating expenditure per tonne of $CO_{2}$ captured and the corresponding energy ratio for the sequestration of 8.05MW power in 4.5MT of tailings, considering distinct permeabilities under a variable re-piping period scenario: (a) $K_{t}=10^{-9}{\;m}^{2}$, (b) $K_{t}=10^{-10}{\;m}^{2}$, (c) $K_{t}=10^{-11}{\;m}^{2}$, (d) $K_{t}=10^{-12}{\;m}^{2}$, (e) $K_{t}=10^{-13}{\;m}^{2}$, and (f) $K_{t}=10^{-14}{\;m}^{2}$. (Operating expenditures (solid lines, left axis) and energy ratios (dashed lines, right axis) depicted for varying reactivity indexes of the tailings, distinguished by different colors in each graph.). (For interpretation of the references to color in this figure legend, the reader is referred to the Web version of this article.)

To illustrate this approach, the quantity of available tailings and the average power demand of the mine were adapted from the previous analysis shown in Fig. 11. The target was set to decarbonize 12.4% (1MW), 37.3% (3MW), and 100% (8.05MW) of the average power demand with an initial layering of 4.5MT of tailings. A wide range was considered for RI, ranging from 0.05% to 10%. The cost model was run to estimate the operating expenditures and the energy ratio for each scenario selected for the variance of the permeability. The results are categorized according to the permeability values and shown in Fig. 12(a–f), Fig. 13(a–f), and Fig. 14(a–f), respectively, for 1MW, 3MW, and 8.05MW of power sequestrated. In all three figures, for $K_{t}=10^{-09}{\;m}^{2}$ and $10^{-10}{\;m}^{2}$, the ${ox}_{t}$ progressively decreased as $R_{wh}$ increased. As in such high permeable tailings, due to low IPL, the ${ox}_{t}$ was mostly contributed by ${ox}_{piping}$. Also, as the lower values of RI required more frequent installation of pipes, higher $R_{wh}$ helped the operation to continue with fewer number of pipes (or blocks). That was why the ${ox}_{t}$ kept on decreasing with the increase of $R_{wh}$. For these two permeabilities, $R_{Ε}$ was always below or close to 1% for all configurations investigated. In all cases (regardless of the permeabilities), considering the re-piping period as a variable in the calculation revealed a clear trend: higher reactivity index (RI) values corresponded to lower values of ${ox}_{t}$.

From $K_{t}=10^{-11}{\;m}^{2}$, the impact of the energy cost was observed to be influence the ${ox}_{t}$, and the $R_{wh}$ associated with the minimum ${ox}_{t}$ started to decrease. RI=0.75% cases can be discussed here as an example. The minimum ${ox}_{t}$ was 11C$, 23C$, and 41C$, for the sequestration of 1MW, 3MW, and 8.05MW of power respectively, as depicted in Fig. 12(c)–. 13(c) and 14(c). The associated $R_{wh}$ with these minimum ${ox}_{t}$ were 70, 50, and 41. The variance in the operating cost per tonne of CO_2_ sequestrated observed here was due to the different re-piping periods and associated piping costs involved. For the 1MW case, in every 4years of operation, a new layer of 4.5MT of tailings was needed along with a new set of injection pipes, whereas for 3MW and 8.05MW cases, the re-piping period shrank to 1.6, and 0.5years, respectively. Also, to balance TPL and IPL ratio, it was necessary to employ larger-sized injection pipes where injection flow rates were higher (higher power sequestration cases). This analysis was analogous to all the other cases examined here. In Fig. 12(d), the minimum ${ox}_{t}$ for each case was accomplished when $R_{wh}$ ranged between 8 to 50. This range shrunk to 8 to 33, and 8 to 27, respectively, in Figs. 13(d) and Fig. 14(d).

Another trend can also be seen in all the results shown in Figs. 12, Figure 13, and Fig. 14. The $R_{wh}$ responsible for the minimum ${ox}_{t}$ in any design configuration decreased with the increase of RI. The results shown in Fig. 14(e) can be discussed here for further explanation. The $R_{wh}$ leading to the minimum ${ox}_{t}$, was 8, for RI=0.5%. It decreased to 2 when RI increased by 10 times. As with a higher reactivity index, the injection pipes will need to be installed less frequently, the ${ox}_{t}$ became dominated by the ${ox}_{elec}$, and the optimum design point was found with a relatively smaller $R_{wh}$.

In the cases with lower permeabilities, designing the system with low $R_{wh}$ can be recommended as it can keep the $R_{e}$ under a reasonable value. For instance, in Fig. 13(f), increasing $R_{wh}$ above 4, would make the $R_{e}$ higher than 20%. However, the percentage of the total power that was being sequestrated was also essential to consider in making the final decision on choosing a suitable operating point.

**Fig. 15:**
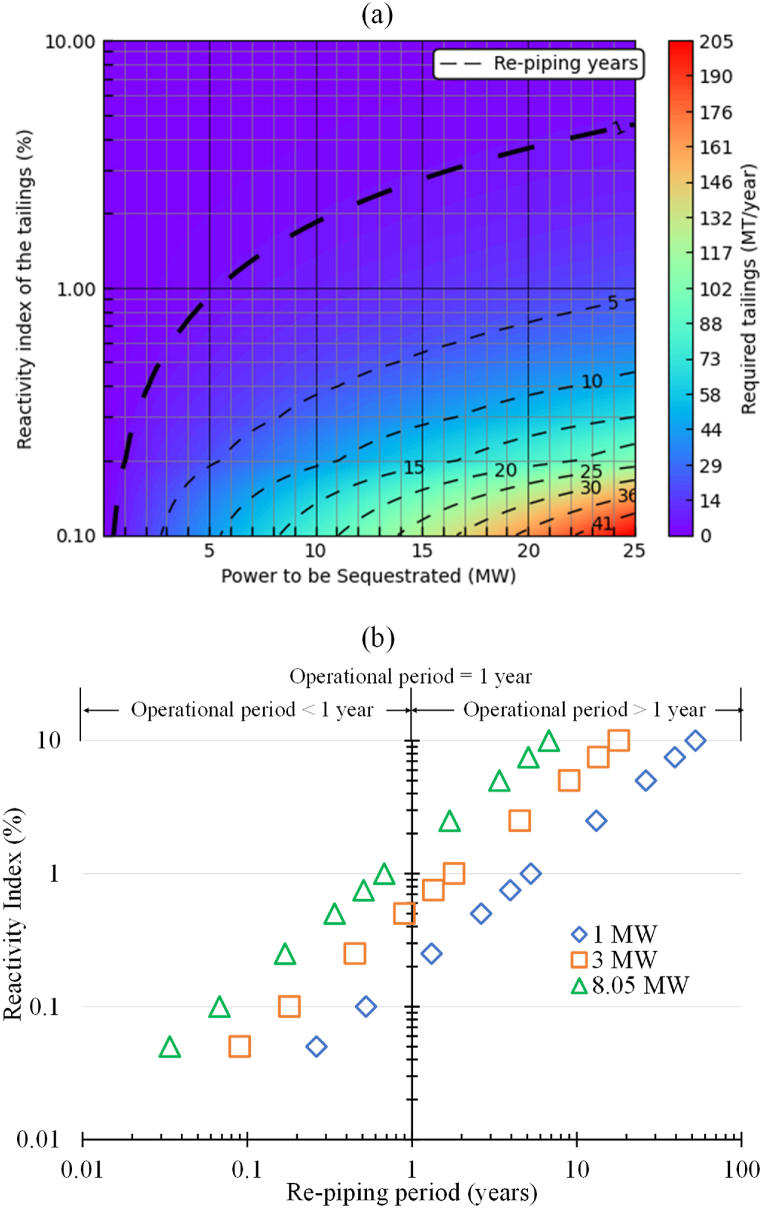
(a) Contour plot showing tailings quantity required for sequestering specific power levels, considering tailings reactivity, and dashed lines representing the re-piping period with an annual availability of 4.5MT of tailings. (b) Re-piping periods for the carbon sequestration cases targeting to capture 1, 3, and 8.05MW power for cases considering 4.5MT of tailings available yearly.

It was important to compile the results presented in Figs. 12, Figure 13, and Fig. 14 to show how variance of the re-piping period in different operating constraints that could impact the decision-making process. A correlation between the available quantity of tailings and the amount of power that could potentially be sequestrated within it, contingent on the reactivity index of the tailings, was visually depicted in Fig. 15(a) using colour-contour representations. The contour map serves as a guide for estimating the potential power that can be decarbonized through flue gas injection into the mine waste, considering the quantity of available tailings in a mining operation, and categorizing them based on their reactivity potential. In the same figure, re-piping periods are also shown by using dashed lines, considering 4.5MT of tailings available annually to commence the operation. Based on the information presented in this figure, achieving power sequestration of 10MW with 4.5MT of tailings (over a re-piping period of 1year) through flue gas injection necessitates that the tailings possess a reactivity index of 2%. The dashed lines representing the re-piping period in Fig. 15(a) will vary depending on the initially available quantity of tailings. Nevertheless, the colour-contour map will remain unchanged, serving as a consistent general reference.

The re-piping periods for all the cases presented in Figs. 12, Figure 13, and Fig. 14 were plotted in Fig. 15(b) against the reactivity index of the tailings. For the 1MW scenarios examined in this study, any reactivity index (RI) exceeding 0.25% resulted in an operational (re-piping) period extending beyond 1year. The RI had to be above 0.75% and 2.5%, for the 3 and 8.05MW cases tested here, respectively, for the re-piping period to be above 1year.

## 4 Conclusion

The reduced-order model established here showed potential to be included in designing a pressure and energy-optimum scenario while targeting to capture the carbon in mine tailings by injecting the diesel exhaust from the power plant. The techno-economic cost model was developed to evaluate the expenses associated with carbon sequestration, considering factors such as piping, and energy operational expenditures. The meticulous consideration of the intricate factors like piping infrastructure and operational energy expenditures shaped this study to be used in the decision-making to assess the operating expenses involved in carbon sequestration in mine tailings through flue gas injection.

Tailings possessing higher permeabilities exhibited reduced energy consumption for operation, and the operating cost was primarily driven by the piping costs. Conversely, in the case of low-permeable tailings, energy expenses played a more significant role in the total operating cost. Striking the right balance between power costs and piping expenditures is pivotal for successful implementation of the proposed concept at a larger scale. This study can be incorporated in the decision-making processes aiming at identifying the optimal operating zone. The findings showed that the operating expenditure of carbon capture within mine tailings can effectively be managed under the threshold of 100C$ per tonne of $CO_{2}$ sequestrated considered in this study. These findings underscored the potential for meaningful greenhouse gas reduction and reinforced the practicality of large-scale adaptation of carbon sequestration strategies in mine tailings within industrial frameworks. The present research demonstrated that through efficient design decisions, the energy requirements for the entire sequestration process can be maintained under 1% of the power sequestrated. The targeted energy ratio is an operating decision, and the threshold for it should be adjusted accordingly to the targeted amount of $CO_{2}$ that needs to be sequestrated. The cost of tailings handling was not included in the model, considering the application in the dry-stack tailings facility. However, the future will focus more on the entire tailings management process to capture $CO_{2}$. Additionally, a thorough life cycle analysis of the entire operation will be vital to maximize the positive outcomes of the proposed concept.

**Funding**

This research was funded by the De Beers Group through the CarbonVault Program with matching funds from Natural Resources Canada (Clean Growth Program, grant number CGP-17-0739).

**Data availability statement**

The data that support the findings of this study are available upon reasonable request.

## CRediT authorship contribution statement

**Durjoy Baidya:** Writing – original draft, Visualization, Validation, Software, Methodology, Investigation, Formal analysis, Data curation, Conceptualization. **Gregory Dipple:** Writing – review & editing, Supervision, Resources, Funding acquisition. **Seyed Ali Ghoreishi-Madiseh:** Writing – review & editing, Supervision, Project administration, Methodology, Conceptualization.

## Declaration of competing interest

The authors declare that they have no known competing financial interests or personal relationships that could have appeared to influence the work reported in this paper.

## References

[1] Schmalenbach K. (2023). Corporate Liability for Transboundary Environmental Harm.

[2] Cf O. (2015).

[3] Hansen J., Sato M., Ruedy R. (Sep 11 2012). Perception of climate change. Proc. Natl. Acad. Sci. U. S. A..

[4] Gasser T., Guivarch C., Tachiiri K., Jones C.D., Ciais P. (Aug 3 2015). Negative emissions physically needed to keep global warming below 2 degrees C. Nat. Commun..

[5] Jacobs A.D., Hitch M. (2011). Experimental mineral carbonation: approaches to accelerate CO2sequestration in mine waste materials. Int. J. Min. Reclamat. Environ..

[6] Southam G. (Mar 1 2020). Accelerating mineral carbonation in ultramafic mine tailings via direct CO2 reaction and heap leaching with potential for base metal enrichment and recovery. Econ. Geol..

[7] Kelemen P., Benson S.M., Pilorgé H., Psarras P., Wilcox J. (Nov 15 2019). An overview of the status and challenges of CO2 storage in minerals and geological formations. Frontiers in Climate.

[8] Power I.M., Dipple G.M., Bradshaw P.M.D., Harrison A.L. (Mar 2020). "Prospects for CO2 mineralization and enhanced weathering of ultramafic mine tailings from the Baptiste nickel deposit in British Columbia, Canada,". Int. J. Greenh. Gas Control.

[9] Levesque M., Millar D., Paraszcza J. (Dec 1 2014). "Energy and mining - the home truths,". J. Clean. Prod..

[10] Ghoreishi-Madiseh S.A., Sasmito A.P., Hassani F.P., Amiri L. (Jan 1 2017). Performance evaluation of large scale rock-pit seasonal thermal energy storage for application in underground mine ventilation. English), Applied Energy.

[11] Froese S., Kunz N.C., Ramana M.V. (2020). Too small to be viable? The potential market for small modular reactors in mining and remote communities in Canada. Energy Pol..

[12] Baidya D., Wynands E., Samea P., Ghoreishi-Madiseh S.A., Dipple G. (Jul 2023). A reduced-order fluid flow model for gas injection into porous media: for application in carbon sequestration in mine tailings. English), Minerals-Basel.

[13] Harrison A.L., Power I.M., Dipple G.M., Mayer K.U. (2011). https://www.engineeringvillage.com/share/document.url?mid=grf_569974f5149e7608208M7ce310178163125&database=grf.

[14] Wilson S.A., Barker S.L.L., Dipple G.M., Raudsepp M., Fallon S.J. (2009). https://www.engineeringvillage.com/share/document.url?mid=grf_8cd50a914856a699b7M364b10178163244&database=grf.

[15] Wilson S., Barker S.L., Dipple G.M., Atudorei V. (Dec 15 2010). Isotopic disequilibrium during uptake of atmospheric CO2 into mine process waters: implications for CO2 sequestration. Environ. Sci. Technol..

[16] Bea S.A., Mayer K.U., Wilson S.A., Dipple G.M. (2011). https://www.engineeringvillage.com/share/document.url?mid=grf_1b5ca36014856aaff63M505810178163244&database=grf.

[17] Assima G.P., Larachi F., Beaudoin G., Molson J. (Jan 2013). Dynamics of carbon dioxide uptake in chrysotile mining residues - effect of mineralogy and liquid saturation. Int. J. Greenh. Gas Control.

[18] Harrison A.L., Power I.M., Dipple G.M. (Jan 2 2013). Accelerated carbonation of brucite in mine tailings for carbon sequestration. Environ. Sci. Technol..

[19] Hills C.D., Tripathi N., Carey P.J. (Jul 14 2020). Mineralization technology for carbon capture, utilization, and storage. Front. Energy Res..

[20] Fricker K.J., Park A.H.A. (Nov 26 2014). Investigation of the different carbonate phases and their formation kinetics during Mg(OH)slurry carbonation. Ind. Eng. Chem. Res..

[21] Stokreef S., Sadri F., Stokreef A., Ghahreman A. (Jun 2022). Mineral carbonation of ultramafic tailings: a review of reaction mechanisms and kinetics, industry case studies, and modelling. Cleaner Engineering and Technology.

[22] Smith P. (2015). Biophysical and economic limits to negative CO2 emissions. Nat. Clim. Change.

[23] Baidya D., de Brito M.A.R., Sasmito A.P., Scoble M., Ghoreishi-Madiseh S.A. (Jul 10 2019). Recovering waste heat from diesel generator exhaust; an opportunity for combined heat and power generation in remote Canadian mines. J. Clean. Prod..

[24] Baidya D., de Brito M.A.R., Ghoreishi-Madiseh S.A. (Sep 1 2020). Techno-economic feasibility investigation of incorporating an energy storage with an exhaust heat recovery system for underground mines in cold climatic regions. Appl. Energy.

[25] Baidya D., de Brito M.A.R., Sasmito A.P., Ghoreishi-Madiseh S.A. (Jan 2022). Diesel generator exhaust heat recovery fully-coupled with intake air heating for off-grid mining operations: an experimental, numerical, and analytical evaluation. Int. J. Min. Sci. Technol..

[26] Kaviany M. (2012).

[27] Nield D.A., Bejan A. (2006).

[28] Ho C.K., Webb S.W. (2006).

[29] Brown G.O. (2002). Proceedings of the Environmental and Water Resources History.

[30] Economides M.J. (2013).

[31] Wang J.Y. (Apr 15 2011). Theory of flow distribution in manifolds. Chem. Eng. J..

[32] Kulkarni A.V., Roy S.S., Joshi J.B. (2007). Pressure and flow distribution in pipe and ring spargers: experimental measurements and CFD simulation - ScienceDirect. Chem. Eng. J..

[33] Wang J.Y., Gao Z.L., Gan G.H., Wu D.D. (Sep 15 2001). Analytical solution of flow coefficients for a uniformly distributed porous channel. Chem. Eng. J..

[34] Amiri L., de Brito M.A.R., Baidya D., Kuyuk A.F., Ghoreishi-Madiseh S.A., Sasmito A.P., Hassani F.P. (Oct 15 2019). Numerical investigation of rock-pile based waste heat storage for remote communities in cold climates. Appl. Energy.

[35] Ghoreishi-Madiseh S.A., Kuyuk A.F., de Brito M.A.R., Baidya D., Torabigoodarzi Z., Safari A. (Feb 2 2019). Application of borehole thermal energy storage in waste heat recovery from diesel generators in remote cold climate locations. Energies.

[36] Bonnemains D., Carlut J., Escartín J., Mével C., Andreani M., Debret B. (Aug 2016). Magnetic signatures of serpentinization at ophiolite complexes. G-cubed.

[37] Lupo J., Hall J. (2010). Proceedings Fourteenth International Conference on Tailings and Mine Waste.

[38] Nwt C., Johnson D.D., Pilotto D. (2017). https://www.miningdataonline.com/reports/Gahcho_Kue_Technical_Report_03162018.pdf.

